# Perks of Early Physical Therapy Rehabilitation for a Patient With Diffuse Axonal Injury

**DOI:** 10.7759/cureus.30886

**Published:** 2022-10-30

**Authors:** Shivani S Lalwani, Ragini Dadgal, Pallavi Harjpal, Sakina S Saifee, Tasneem M Lakkadsha

**Affiliations:** 1 Department of Physiotherapy, Ravi Nair Physiotherapy College, Datta Meghe Institute of Medical Science, Wardha, IND

**Keywords:** rehabilitative approaches, hemiplegia, glasgow coma scale, conservative rehabilitation, diffuse axonal injury, case report, quality of life, rehabilitation, physiotherapy, traumatic brain injury

## Abstract

Traumatic brain injury (TBI) is characterized as any neurological trauma that develops after birth and therefore is completely unconnected from congenital anomalies, developmental disorders, or gradual processes. People who have survived accidents or other severe head injuries that left them with brain damage have been linked to memory loss and disability. On February 9, 2022, a 23-year-old individual was taken to a nearby hospital with a head injury after being involved in a traffic accident that morning while under the influence of alcohol. After several tests, the individual was identified as having a diffuse axonal injury in the anterolateral aspect of the pons, which was confirmed by an MRI and CT scan of the brain. The patient had been managed conservatively with appropriate medications like (tab Zifi® 200mg, tab Epilive® 500mg, tab Strocit plus®, tab Modalert®, tab Oxynerve plus®, etc.) along with physiotherapy, and other supportive treatments. Key indicators involve recovery of consciousness, normalization of muscle tone, earlier onset of movements, adequate strength, and quality of life. The TBI rehabilitation service is advantageous, as supported by proportionally massive progress in exercise tolerance and overall health. The above case study serves as an example of an extensive rehabilitation program for patients who have undergone conservative treatment after suffering a diffuse axonal injury.

## Introduction

Traumatic brain injury (TBI) is characterized as any neurological trauma that develops after birth and therefore is completely unconnected to congenital anomalies, developmental disorders, or gradual processes [1]. TBI is widespread, affecting fifty million people annually, and therefore is linked to impaired cognition and dementia in people who survive it [2]. One of the most frequent and disturbing forms of TBI is diffuse axonal injury (DAI), which is also a significant contributor to a consistent vegetative state as well as loss of consciousness following serious brain injury. It does not result from secondary changes like oxygen deprivation, neurological damage, or increased intracranial tension; rather, it happens as a result of the influence of external pressures like acceleration and deceleration forces that act just at the time of the trauma [3].

DAI is well-known to be among the most significant risk factors for mortality and comorbidities in TBI patients. This could lead to physiological, intellectual, and behavioral problems that lower well-being, make people disabled, and interfere with daily practices. Even though brain tissue is functionally compromised but not destroyed, if therapy is initiated as soon as possible, the brain could progressively gain back its functional capacity and the person's neural function can be restored [4].

In order to help people recover, physical therapists have employed a wide variety of techniques for people who have been affected by TBI, and they concentrate on particular restorative ideas supported by an independent physiotherapist [5]. The main therapeutic objectives are often the restoration of awareness as well as sensorimotor perspective, the protection from secondary damage like pneumonia or contractures, as well as muscle strengthening. The main objective is to attain the highest level of mobility as well as confidence in terms of self-care [6].

We report the case of a 23-year-old man who survived a diffuse axonal injury and required impactful physical therapy care to hasten healing by attempting to prevent or try to resolve postoperative problems as well as offering rehabilitation and support to assist the patient in returning to their normal healthy state. Important indicators consist of recovery of consciousness, normalization of muscle tone, earlier onset of movements, adequate strength along with life quality.

## Case presentation

Patient information

On February 9, 2022, a 23-year-old man was brought to our emergency unit with a head injury after being involved in a road traffic accident that morning while under the influence of alcohol. At post-evaluation, the Glasgow Coma Scale (GCS) score of the patient was less than 7 (eye-opening score - 2, verbal response - 2, motor response - 2), so he was moved to the Neurosurgery Intensive Care Unit (NICU), where due to his lack of consciousness he was intubated and placed on Intermittent Mandatory Ventilation (IMV). Following a series of tests, on February 10, 2022, the patient was diagnosed with a diffuse axonal injury in the anterolateral aspect of the pons, which was confirmed by an MRI and CT scan of the brain. The MRI study also revealed contusion with oedema in the midbrain and the left gangliocapsular region.

With the assistance of medications, physiotherapy, and other supportive treatments, the patient was entirely managed conservatively. On February 17, 2022, the patient was extubated as he was able to maintain his saturation level in the ICU and tracheostomised to obtain long-term airway protection. The patient was shifted to the neurosurgical ward on February 23, 2022, as he was vitally, clinically, and neurologically stable. Since he was recovering well, a decannulation trial was performed, which was successful. On March 8, 2022, he was discharged with advice to continue medical management and physiotherapy.

Clinical findings

Clinical Assessment in the ICU

The clinical examination was performed on post-admission day four, i.e., February 12, 2022, with the attending doctor and patient's relative's agreement. He was positioned in a supine position with his head end elevated to 30 degrees. A Ryles tube and a Foleys catheter were noticed during the inspection. On examination, the patient had a GCS score of 6. According to Rancho Los Amigos Levels of Cognitive Functioning (RLA-LOCF), the patient was showing a generalized response. The Mini-Mental State Examination (MMSE) score was 0. A tonal examination showed normal tone in the lower and upper limbs of the left side, but the lower and upper limbs of the right side had no tone.

Clinical Assessment in the Ward

The patient had been examined while sitting comfortably in the ward with proper back support. The patient’s GCS score was 10. According to RLA-LOCF, the patient was showing a localized response. The MMSE score was 5. The Modified Ashworth Scale (MAS) rating of 1 for the right side lower and upper limbs indicated a mild gain in muscular tone, which was exhibited with catch and release during the tonal examination. The left side’s lower and upper limbs had normal tone.

Clinical Assessment in the Physiotherapy Rehab

The patient had been examined while sitting comfortably with proper back support in the rehabilitation centre. The patient’s GCS score was 11. According to RLA-LOCF, the patient was showing a confused and agitated response. The MMSE score was 10. The lower and upper limbs of the left side had normal tone, as determined by a tonal examination, but the right side's lower and upper limbs had a mild gain in muscular tone exhibited with catch, as indicated by a Modified Ashworth Scale (MAS) score of 1+.

Therapeutic interventions

The objective of rehabilitation for an individual would have aimed to assist him to resume his routine day-to-day chores with the least amount of complexity. Table 1 illustrates the patient’s physical therapy care in the neurosurgery ICU. Table 2 illustrates the patient’s physical therapy care in the neurosurgery ward. Table 3 illustrates the patient’s physical therapy care in the physiotherapy rehab. 

**Table 1:** The Patient’s Physical Therapy Care in the Neurosurgery ICU. PROM: Passive range of motion, ET: Endotracheal tube, PNF: Proprioceptive neuromuscular facilitation, ICU: Intensive care unit

Serial no.	Physical Therapy Objectives	Rehabilitative Approaches
1)	To make them aware of the condition of the patient as well as gain the cooperation and approval of caregivers and family.	Guidance as well as counselling for family and caretakers on the value of adhering to a workout schedule.
2)	To prevent pressure sores from prolonged immobilization.	1) Manual positioning every 2 hours. 2) Air bed provided.
3)	To preserve joint function as well as movement while avoiding joint rigidity.	Bilateral PROM exercises for the upper and lower extremities.
4)	To assist in clearing the airways.	1) Manual Vibrations & Percussions of the Chest. 2) Oral and ET tube suctioning. 3) Chest PNF – End expiratory pressure.
5)	To normalize the muscle tone of the right upper and lower extremities.	Facilitatory techniques: 1) Quick Icing 2) Tapping 3) Quick Stretch

**Table 2:** The Patient’s Physical Therapy Care in the Neurosurgery Ward. ICU: Intensive care unit, AROM: Active range of motion.

Serial no.	Physical Therapy Objectives	Rehabilitative Approaches
1)	To make them aware of the condition of the patient as well as gain the cooperation and approval of caregivers and family.	Guidance as well as counselling for family and caretakers on the value of adhering to a workout schedule.
2)	To preserve chest movement and increase lung capacity and volume.	Exercises for the expansion of the thorax: flexion of the shoulder with a deep inhalation and exhalation while extension.
3)	To preserve joint function as well as movement while avoiding joint rigidity.	Bilateral AROM exercises for the lower and upper limbs.
4)	To improve the flexibility of muscles and to prevent tightness.	Stretching for: Hamstrings, tendoarchilles, and adductors.
5)	To resist muscle atrophy and to enhance muscular strength and endurance.	Static and isometric exercises for Quadriceps and hamstrings
6)	To normalize the muscle tone of the right upper and lower extremities.	Facilitatory techniques: 1) Quick Icing 2) Tapping 3) Quick Stretch
7)	To promote mobilization of the patient and to increase the level of alertness.	Out-of-bed mobilization using a wheelchair.

**Table 3:** The Patient’s Physical Therapy Care in the Physiotherapy Rehab. ROM: Range of motion, PNF: Proprioceptive neuromuscular facilitation, FES: Functional electrical stimulation, SLR: Straight leg raise, CIMT: Constrained induce movement therapy

Serial no.	Physical Therapy Objectives	Rehabilitative Approaches
1)	To make them aware of the condition of the patient as well as gain the support and approval of caregivers and family.	Guidance as well as counselling for the patient, family, and caretakers on the value of adhering to a workout schedule.
2)	To initiate muscle contraction (agonist-antagonist activation) of the hemiplegic right side	FES for the right upper and lower limb extensors and flexors. (Figures 1 and 2)
3)	To maintain flexibility of muscles and to prevent tightness.	Stretching for: Hamstrings, tendoarchilles, and adductors.
4)	To resist muscle atrophy and to enhance muscular strength and endurance.	Strength training for the upper and lower extremities -Overhead arm flexion-extension, abduction exercises. -Dynamic Quads -SLR -Thera band strengthening -Weight cuff training (Figure 3)
5)	To strengthen the abdominal muscles and improve trunk balance.	-Static abdominals -Pelvic bridging -Pelvic rotations -Crunches -Bedside sitting with minimal assistance.
6)	To encourage flexibility, strength, and coordination throughout the limb’s entire ROM.	PNF for right upper limb: D1 and D2 flexion-extension patterns.
7)	To stimulate alertness and early weight-bearing on the affected lower extremities.	Verticalization using a Tilt table.
8)	To improve coordination of the patient	-Coordination exercises - Reach-out activities.
9)	To improve arm activity and to regain dexterity and precision of the affected hand.	-CIMT
10)	Balance and postural control training.	With appropriate verbal and mirror feedback, weight shifts and midline orientation activities in sitting and standing in anteroposterior and lateral directions are explored.
11)	Initiate training for gait and locomotion with proper feedback.	Parallel bar activities: (Figure 4) -Weight shifts in standing -Sit-to-stand training -Walking with support -Frenkel’s exercise for single-leg stance -Step up and step-down training
12)	Complex gait training	-Obstacle walking. -Walking on different surfaces.

**Figure 1:**
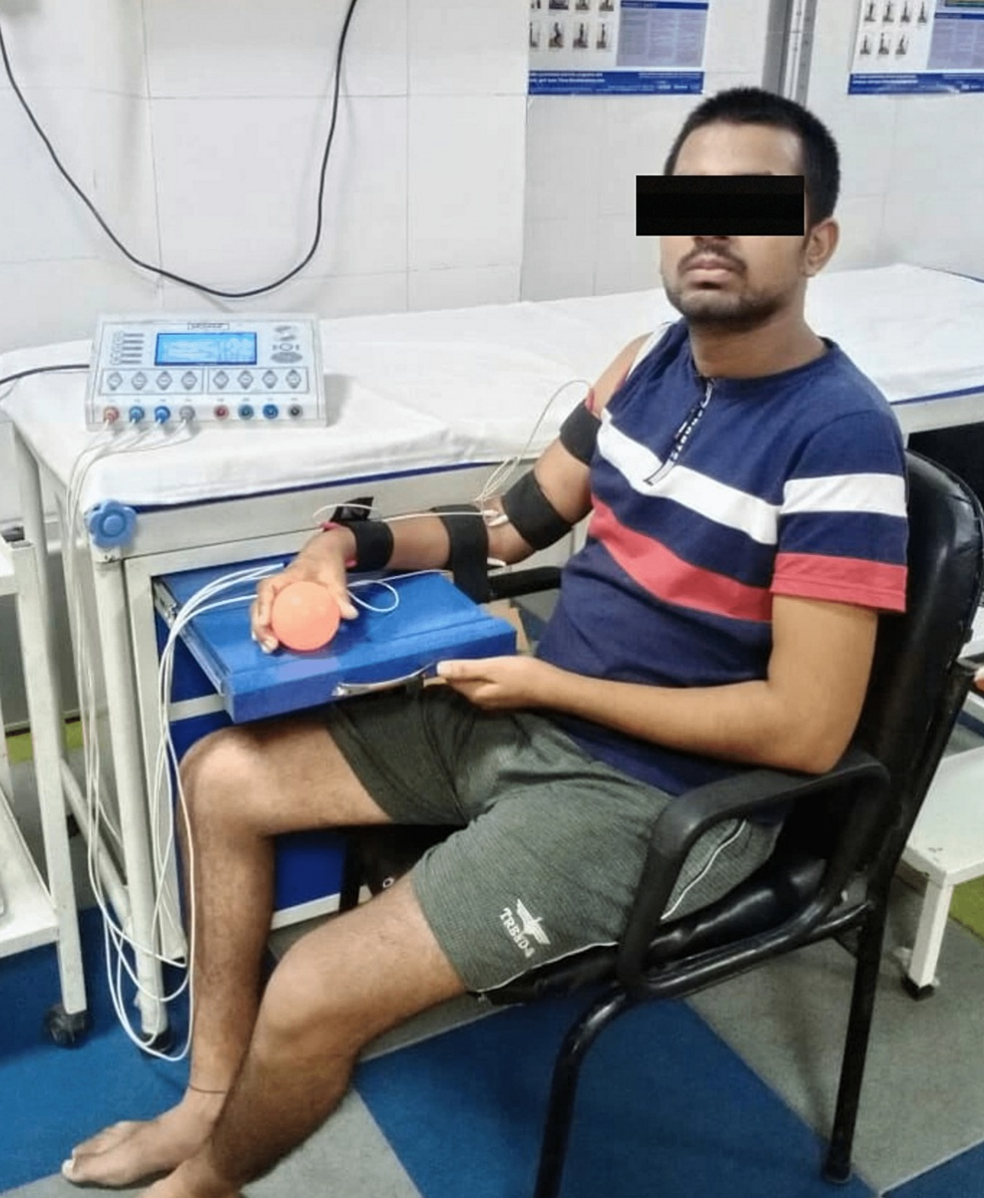
Displays Functional Electrical Stimulation for Flexors and Extensors of the Right Upper Limb Provided to the Patient in Physiotherapy Rehab.

**Figure 2:**
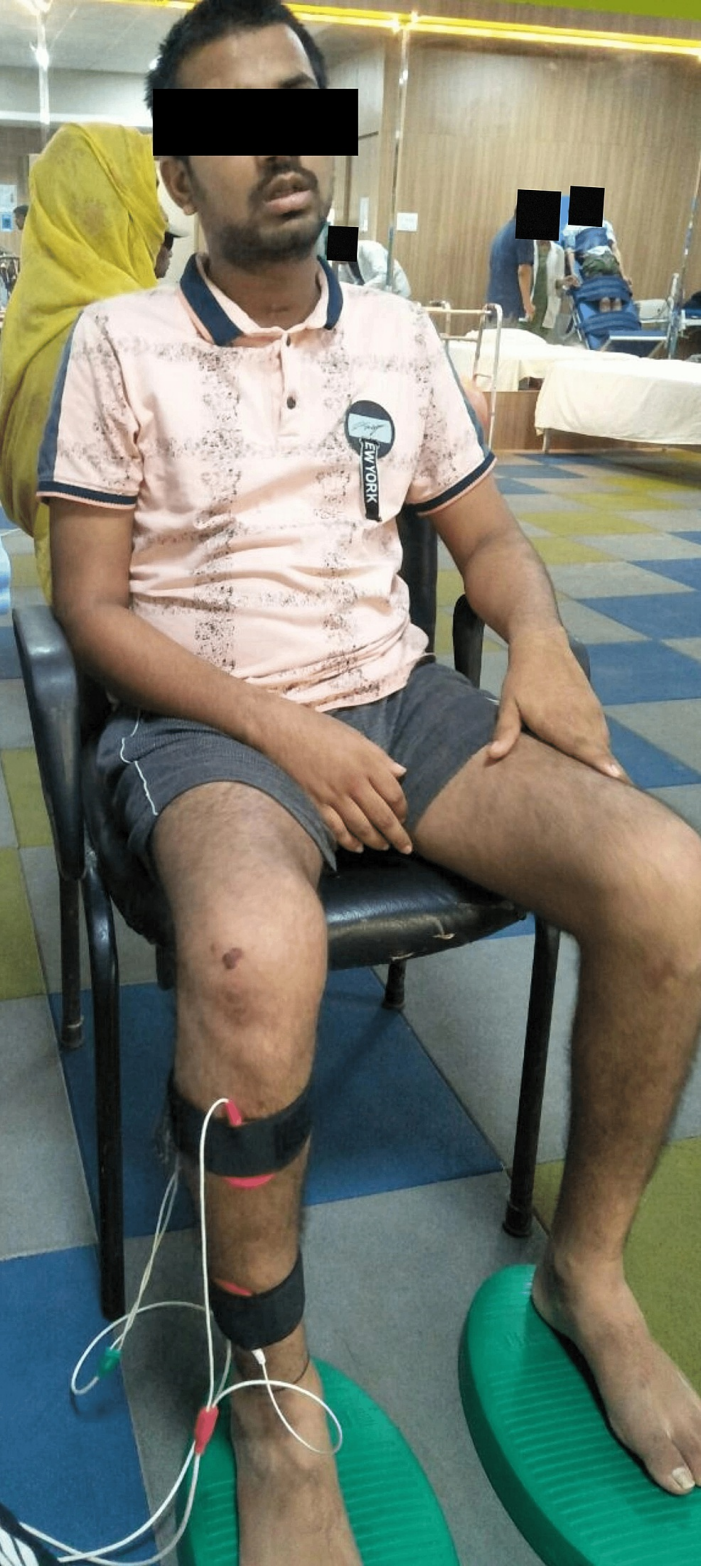
Displays Functional Electrical Stimulation for Flexors and Extensors of the Right Lower Limb Provided to the Patient in Physiotherapy Rehab.

**Figure 3:**
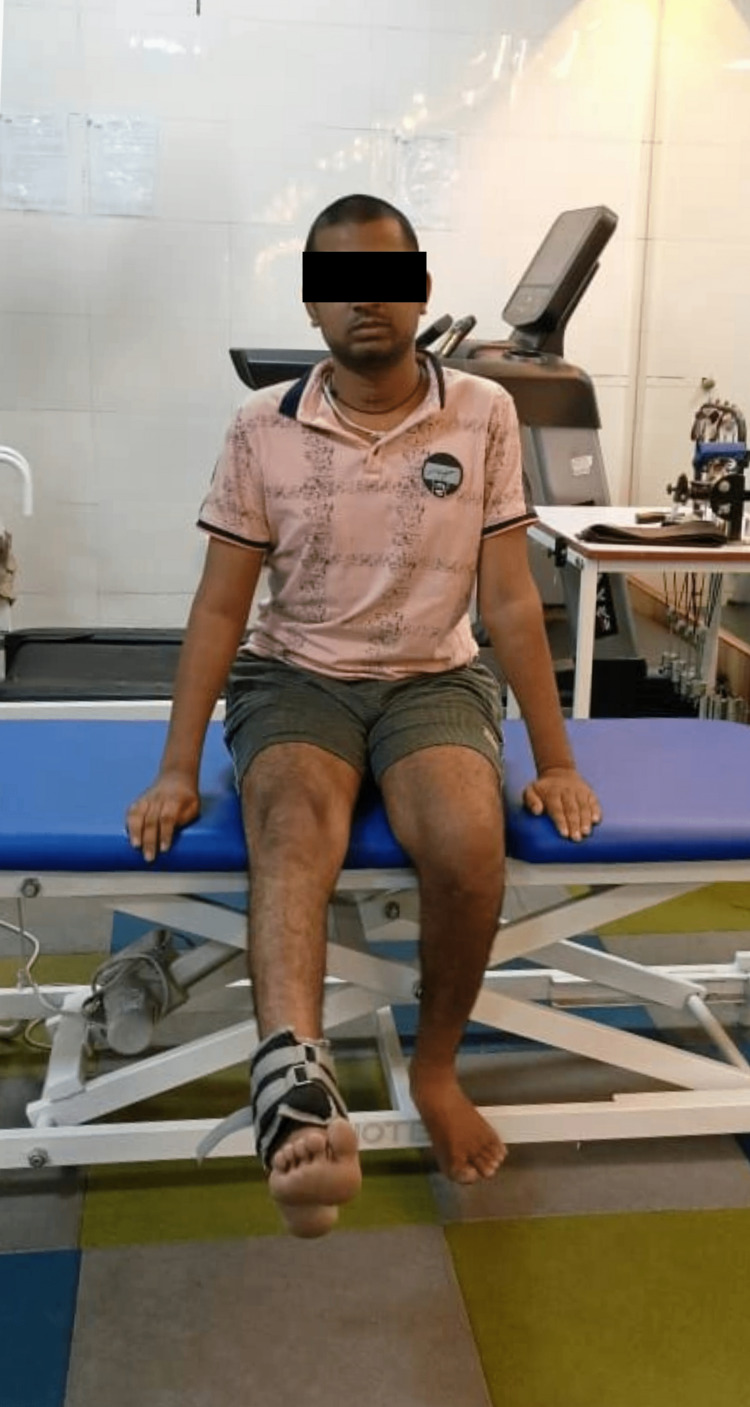
Resisted Exercises Provided to the Patient in Physiotherapy Rehab. The Patient is Performing Dynamic Quads Using a Weight Cuff.

**Figure 4:**
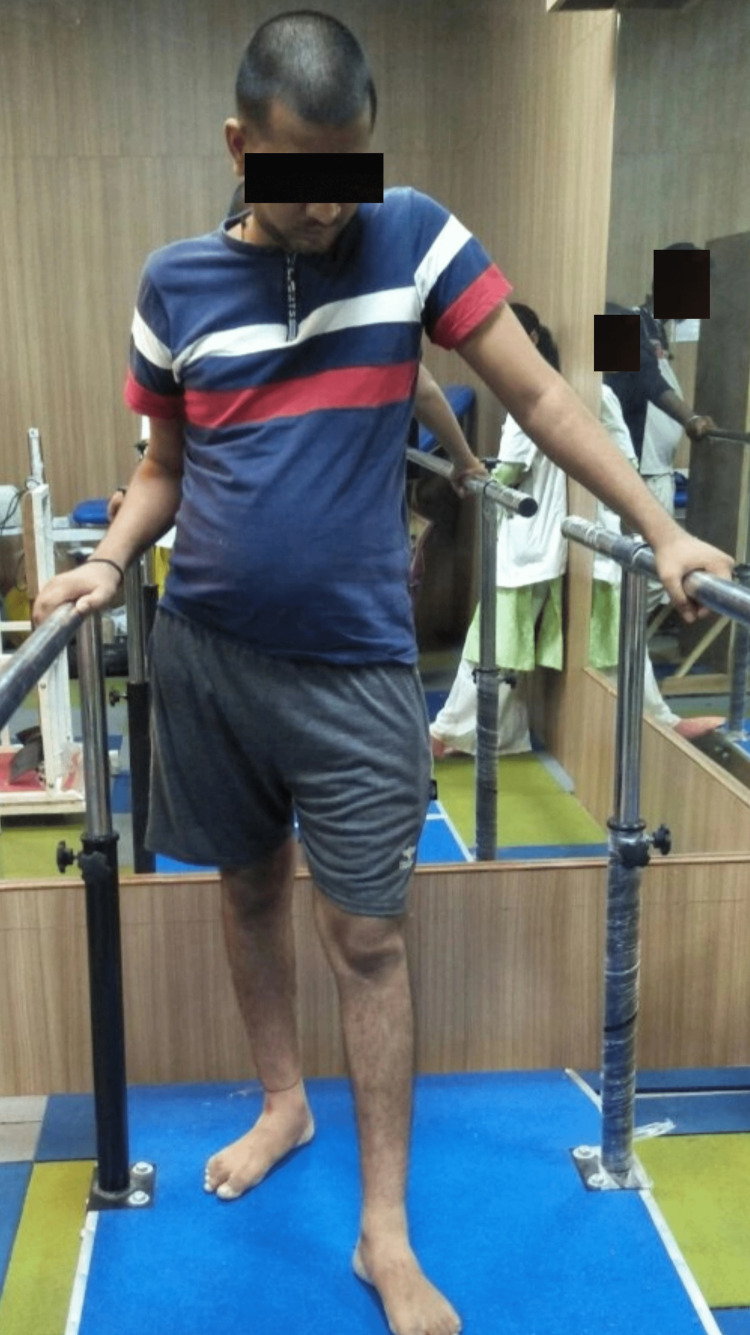
During Physical Therapy Rehabilitation, the Patient Received Gait Training With the Aid of a Parallel Bar

Figure 1 displays functional electrical stimulation for flexors and extensors of the right upper limb. Figure 2 displays functional electrical stimulation for flexors and extensors of the right lower limb. Figure 3 shows the resisted exercises provided to the patient in physiotherapy rehab. During physical therapy rehabilitation, the patient received gait training with the aid of a parallel bar (Figure 4).

The outcome of rehabilitation approaches and follow-up

Following physical therapy rehabilitation, before and after rehabilitation overall results were assessed. Table 4 displays the scores of outcome indicators.

**Table 4:** Measures of Rehabilitation Outcomes Before and After Rehabilitation. GCS: Glasgow coma scale, RLA-LOCF: Rancho Los Amigos Level of cognitive functioning, MAS: Modified Ashworth Scale, MMSE: Mini-mental state examination, MMT: Manual Muscle Testing, BBS: Bergs Balance Scale, FIM: Functional Independence Measure.

Serial. no.	Outcomes measured	Pre-physical therapy rehabilitation result	Post-physical therapy rehabilitation result
1.	GCS	11/15	15/15
2.	RLA-LOCS	Confused agitated response	Automatic appropriate response
3.	MAS	1+/4	0/4
4.	MMSE	10/30	27/30
5.	MMT of shoulder muscles	Score (On a scale of 5)	Score (On a scale of 5)
	Abductors	1	4
	Adductors	1	4
	Flexors	1	4
	Extensors	1	4
6.	Hip muscles MMT	Score (On a scale of 5)	Score (On a scale of 5)
	Flexors	1	4
	Extensors	1	4
	Abductors	1	4
	Adductors	1	4
7.	BBS	0/56	48/56
8.	FIM	1/7	5/7

## Discussion

The patient, in this case, had a severe diffuse axonal injury, which had been managed conservatively with appropriate medications, physiotherapy, and other supportive treatments. Three levels of physical therapy care were offered, starting in the intensive care unit, moving on to the ward, and concluding with physiotherapy rehab. The objectives of the rehabilitation process were established by first completely educating the patient and his family about his condition and the importance of physiotherapy to his recovery.

Soft tissue contractures and changes in muscle tone are two major physical issues that frequently coexist in severe TBI patients. The physiotherapist may occasionally use a variety of splints, personalized orthoses, and plaster casts in conjunction with some other active or passive "stretching" techniques for tissue (such as proper positioning, passive motions, and bearing of weight). Typically, the purpose of treatment is to lengthen shortened soft tissues and/or inhibit/reduce increased muscle tone [7]. So, with this aim, gentle range-of-motion exercises, chest-clearing techniques, and facilitative techniques for restoring muscle tone to normal were all offered in the initial stages of rehabilitation. Further, stretching techniques were provided to improve joint flexibility and prevent muscle tightness, strengthening exercises were given progressively to improve the power and endurance of the muscles.

A significant portion of the physiotherapist's role in serious TBI treatment might be characterized as functional skill acquisition. The brain-injured person needs to rebuild the capacity to carry out functional movement activities, including trying to stand up, move, stair climbing, transfer of position, as well as attempting to reach and clutch. For physiotherapists, these are their main priorities [7]. So, in the later stages of rehabilitation, techniques like PNF (Proprioceptive Neuromuscular Facilitation) for the upper limb, verticalization using a tilt table, coordination exercises, reach-out activities, balance, postural control training, and gait training were provided to the patient.

Rehabilitation following an acquired brain injury could provide essential learning experiences designed to persuade the cortical neuroplasticity processes which are impacted by post-injury behavior [8]. Relearning motor skills and developing new motor skills are important for children and youth with acquired brain injury [8]. Research done by Wade et al. is characteristic of research findings that implicitly base their findings on therapy that uses a "movement science" methodology. Following a short-duration therapy based on "motor learning", the study discovered that there could be substantial gains in moving parameters, functional activities, and sway while taking postures [7].

In accordance with the present study, a skilled neurophysical therapist offered the patient a meticulously planned physiotherapy rehabilitation facility with a wide range of exercises as well as resistive equipment. The primary objective of the physical therapy sessions was to promote individual independence in walking and negligible everyday assistance by improving the activity of the left lower and upper extremities while also preserving the muscle and joint integrity of the right upper and lower limbs. The patient received a written protocol, was told to complete the majority of the exercises at home as part of the program, and was instructed to regularly attend his physiotherapy appointments.

This case report's primary objective was to emphasize the value of physical therapy and rehabilitation for individuals with diffuse axonal injury and to present an extensive framework with profound therapeutic methods for such patients in an attempt to reach their key performance indicators and prognosis. There’s never been any study done previously that offers patients with diffuse axonal injury a full physiotherapy rehabilitation program.

## Conclusions

The post-traumatic brain injury treatment plan is impactful, with remarkable progress in physical as well as functional health. The particular study mentioned above offers a thorough plan for caring for patients who have suffered diffuse axonal injuries. Although the patient did not fully recover during the course of treatment, significant proportions of the recuperative targets had been met, such as enhanced muscular strength, continually rising joint mobility, elevated functional status, pain control, and improvements in the patient's gait and daily activities after several months of targeted physical therapy treatment.
